# Establishment and antitumor effects of dasatinib and PKI-587 in BD-138T, a patient-derived muscle invasive bladder cancer preclinical platform with concomitant EGFR amplification and PTEN deletion

**DOI:** 10.18632/oncotarget.10539

**Published:** 2016-07-12

**Authors:** Nakho Chang, Hye Won Lee, Joung Eun Lim, Da Eun Jeong, Hye Jin Song, Sudong Kim, Do-Hyun Nam, Hyun Hwan Sung, Byong Chang Jeong, Seong Il Seo, Seong Soo Jeon, Hyun Moo Lee, Han-Yong Choi, Hwang Gyun Jeon

**Affiliations:** ^1^ Department of Health Sciences and Technology, SAIHST, Sungkyunkwan University, Seoul 06351, Korea; ^2^ Department of Neurosurgery, Samsung Medical Center, Sungkyunkwan University School of Medicine, Seoul 06351, Korea; ^3^ Institute for Refractory Cancer Research, Samsung Medical Center, Seoul 06351, Korea; ^4^ Institute for Future Medicine, Samsung Medical Center, Seoul 06351, Korea; ^5^ Department of Urology, Samsung Medical Center, Sungkyunkwan University School of Medicine, Seoul 06351, Korea; ^6^ Department of Anatomy and Cell Biology, Samsung Medical Center, Sungkyunkwan University School of Medicine, Seoul 06351, Korea; ^7^ Samsung Advanced Institute of Technology, Samsung Electronics Co., Ltd., Seoul 06351, Korea

**Keywords:** muscle invasive bladder cancer, PTEN, EGFR, drug screening, patient-derived xenograft

## Abstract

Muscle-invasive bladder cancer (MIBC) consists of a heterogeneous group of tumors with a high rate of metastasis and mortality. To facilitate the in-depth investigation and validation of tailored strategies for MIBC treatment, we have developed an integrated approach using advanced high-throughput drug screening and a clinically relevant patient-derived preclinical platform. We isolated patient-derived tumor cells (PDCs) from a rare MIBC case (BD-138T) that harbors concomitant epidermal growth factor receptor (EGFR) amplification and phosphatase and tensin homolog (PTEN) deletion. High-throughput *in vitro* drug screening demonstrated that dasatinib, a SRC inhibitor, and PKI-587, a dual PI3K/mTOR inhibitor, exhibited targeted anti-proliferative and pro-apoptotic effects against BD-138T PDCs. Using established patient-derived xenograft models that successfully retain the genomic and molecular characteristics of the parental tumor, we confirmed that these anti-tumor responses occurred through the inhibition of SRC and PI3K/AKT/mTOR signaling pathways. Taken together, these experimental results demonstrate that dasatinib and PKI-587 might serve as promising anticancer drug candidates for treating MIBC with combined EGFR gene amplification and PTEN deletion.

## INTRODUCTION

Muscle invasive bladder cancer (MIBC) correlates with a high propensity for lymphatic and distant metastasis compared to non-muscle invasive bladder cancer (NMIBC) [1]. Locally advanced and/or metastatic MIBC is associated with a poor prognosis and is difficult to treat even after cystectomy and lymph node dissection with accompanying conventional platinum based chemotherapy [2, 3]. As no truly effective systemic therapies are available for MIBC, there remains a pressing need for novel therapies to improve survival. Notably, NMIBC and MIBC are associated with distinct genotypic and molecular patterns [4, 5]. Previous integrated studies of 131 patients with MIBC identified several key alterations in factors involved in the PI3K/AKT/mTOR, CDKN2A/CDK4/CCND1, and RTK/RAS pathways including ERBB2, ERBB3, and FGFR3 [6]. Another recent report analyzing a series of 35 relapsed bladder cases also found that 83% of the cases had at least one actionable genomic alteration amenable to the application of targeted therapy [7].

Such rational targeting of activating mutations has the potential to enhance the cure rate of MIBC when used in combination with or instead of conventional cytotoxic chemotherapy. With the goal of accelerating the adoption of a precision medicine-based approach for patients with MIBC, molecular stratification of MIBC based on validated predictive biomarkers might identify clinically relevant patient subgroups and help to provide effective targeted therapies. However, the development of successful molecular based therapies in MIBC remains challenging because of the substantial genomic heterogeneity of high-grade MIBCs [8, 9]. In particular, the low response rate is in part attributed to the fact that most cancers harbor multiple genetic aberrations [10, 11]. Such pathway alterations were found to be independently distributed, suggesting that the mutation of two pathway members might have synergistic or additive effects through non-canonical functions.

The analysis of tumor biology by molecular manipulation, identification of relevant predictive biomarkers, and preclinical testing of novel therapeutic agents critically depends on conclusive *in vivo* models of human cancer. However, the correlation of drug sensitivity between conventional cell lines and clinical trials is, in general, poor [12]. These limitations have been resolved by patient-derived xenografts (PDXs) in which specific genetic, molecular and biological characteristics of the original tumor are reliably retained. For example, the establishment of bladder cancer PDXs by grafting representative cancer tissue into the subcutaneous compartment [13, 14] or under the renal capsule of immune-deficient mice [15] has previously been reported.

Herein, we identified an unprecedented locally advanced MIBC case that harbored both epidermal growth factor receptor (EGFR) amplification and phosphatase and tensin homolog (PTEN) deletion. To identify an optimal therapeutic option for this patient, we ascertained drug candidates using *in vitro* high-throughput drug screening (HTS) on patient-derived tumor cells (PDCs) and validated the *in-vivo* efficacy of the identified target molecules in PDXs that recapitulated the genetic, molecular, and histopathological characteristics of the parental tumor.

## RESULTS

### Characterization of *EGFR*-amplified and *PTEN*-deleted MIBC PDCs (BD-138T)

**Figure 1:**
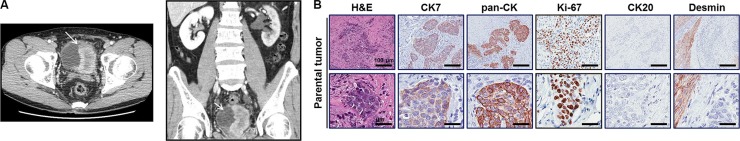
Case presentation of patient 138T with muscle-invasive bladder cancer and validation of patient-derived tumor tissue (**A**) Computed tomography (CT) scan of the pelvis indicates a large bladder tumor invading the base of the bladder of patient 138T. The bladder is visible at the bottom center of the scan. The arrow indicates the tumor region. The tumor tissue appears as cloudy material within the bladder, whereas the remaining area in the bladder is eclipsed. (**B**) Representative images of hematoxylin/eosin (H&E) and immunohistochemistry staining of BD-138T tumor tissue with antibodies for the indicated proteins. CK7, cytokeratin 7; pan-CK, pan-cytokeratin; CK20, cytokeratin 20. Scale bars: 100 μm (Top), 20 μm (Bottom).

BD-138T cells were obtained from a male patient, aged 56 years, previously diagnosed with high-grade transitional cell carcinoma (TCC) in the urinary bladder (Figure 1A). Cystoscopy and transurethral resection of the tumor was conducted; however, residual tumor remained. Pathological examination confirmed a grade 3 urothelial carcinoma with evidence of invasion into the muscular layer (T2G3). As preoperative imaging modalities did not identify any signs of distant metastasis, neoadjuvant chemotherapy was not administered. He received radical cystectomy followed by adjuvant chemotherapy (gemcitabine and paclitaxel). Through final pathological examination, the tumor was staged as a pT3bN1. No signs of local relapse or distant metastasis were detected until the 23 month postoperative follow-up.

**Figure 2:**
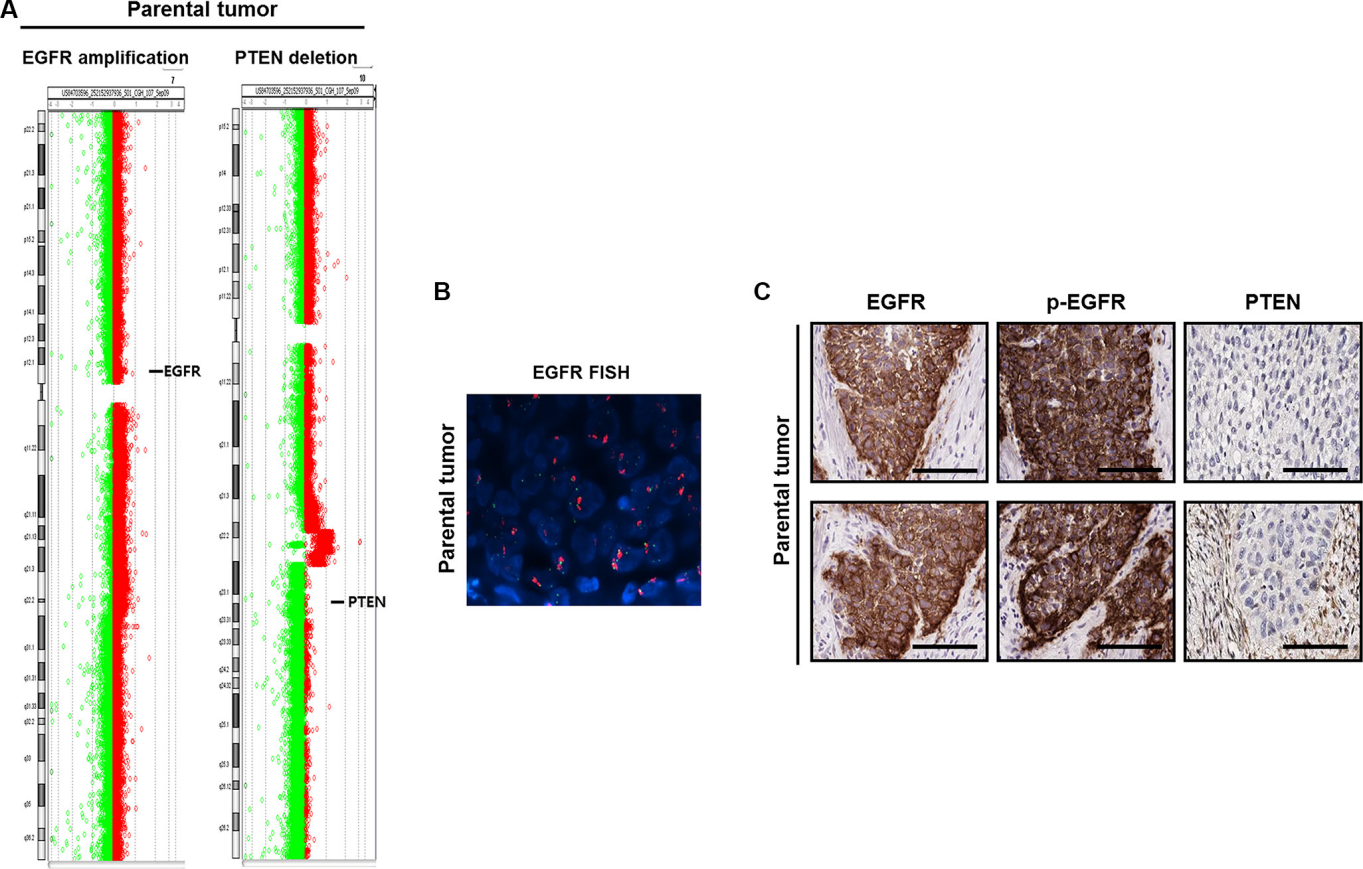
Identification of gene alterations in 138T muscle-invasive bladder cancer (**A**) Comparative genomic hybridization array analysis of the tumor from patient 138T, indicating the mutual *EGFR* amplification and *PTEN* deletion. Individual chromosome ratio plots are shown with red representing amplified regions and green representing deleted regions. High-level *EGFR* amplification in chromosome 7 (left). *PTEN* deletion in chromosome 10 (right). (**B**) Epidermal growth factor receptor (*EGFR*) amplification as assessed by FISH in 138T patient-derived cells (PDCs). Orange signal: *EGFR*; green signal: *CEP7*; blue signal: DAPI counterstaining. (**C**) Representative images of immunohistochemical staining of 138T PDC tumor tissue showing EGFR, p-EGFR (Tyr1068), and PTEN. Scale bars, 100 μm.

Prior to the analysis, we demonstrated the urothelial origin of the tumor cells through hematoxylin-eosin (H&E) staining and immunohistochemistry (IHC) analysis for cytokeratin (CK) 7, pan-CK, Ki-67, CK20, and desmin (Figure 1B) [16, 17]. Next, we analyzed single nucleotide variations, copy number variation, and translocation using genomic DNA via array comparative genomic hybridization (aCGH) and the CancerSCAN^™^ system [18] to identify cancer-driving pathways that might be therapeutically targeted in BD-138T MIBC. Notably, this strategy revealed high-level *EGFR* gene (7p11.2) amplification and bi-allelic *PTEN* gene (10q23.31) deletion in BD-138T patient samples (Figure 2A and 2B). Strong EGFR and p-EGFR expression and the lack of PTEN expression was confirmed by IHC analysis (Figure 2C). In the genomic analysis of 413 bladder cancers with genomic sequence data from The Cancer Genome Atlas (TCGA) (http://www.cbioportal.org), we identified discrete *EGFR* amplification (5%) and *PTEN* deletion (5%); however, cases harboring both EGFR and PTEN alteration as observed in the current MIBC case were extremely rare, suggesting that these two alterations are mutually exclusive.

### Anti-tumor efficacy of dasatinib and PKI-587 in BD-138T MIBC PDCs *in vitro* through anti-proliferative and pro-apoptotic effects

**Figure 3:**
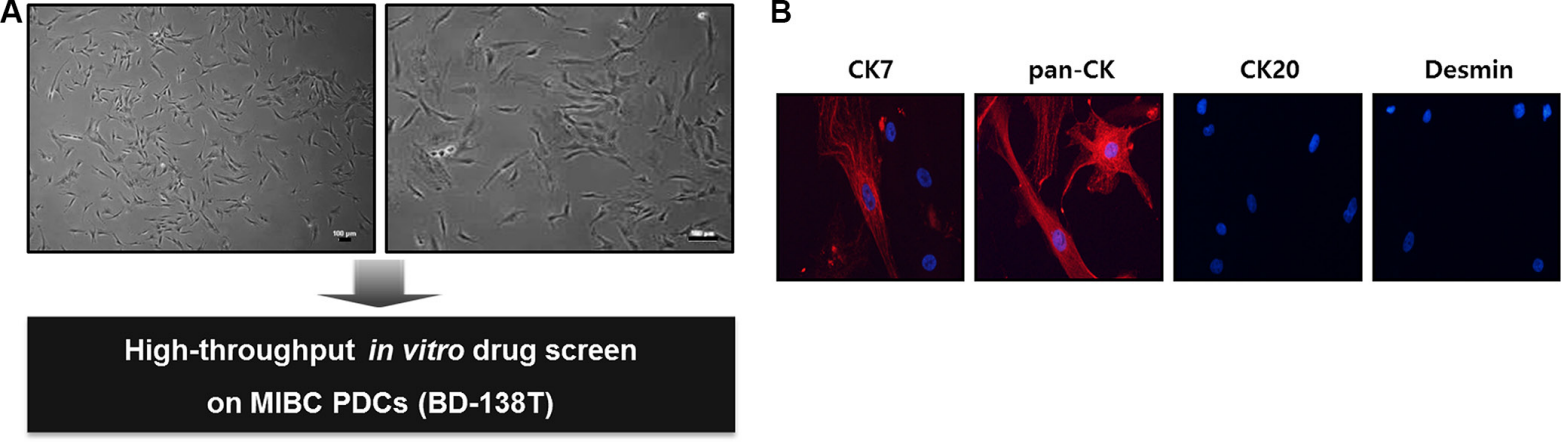
Establishment and validation of patient-derived cells (PDCs) from 138T muscle-invasive bladder cancer (MIBC) (**A**) The establishment of PDCs from 138T MIBC tissues. The tissues were dissociated into single cells for *in-vitro* drug screening using a high-throughput screening (HTS) system. Scale bars: 100 μm. (**B**) Representative confocal microscopy images of immunofluorescence staining of cytokeratin (CK) 7, pan-CK, CK 20, and desmin in 138T PDCs. Scale bars, 100 μm. Red: indicated protein. Blue: DAPI.

To investigate potential anti-cancer drug candidates for BD-138T, we established PDCs from the primary bladder tumor for *in-vitro* drug HTS (Figure 3A). The isolated BD-138T PDCs were positively stained for pan-CK and CK7 whereas they were negatively stained for CK20 and desmin (Figure 3B), confirming that the cells were of urothelial cell origin and that neither fibroblast nor smooth muscle cells were present. The presence of *EGFR* amplification and *PTEN* deletion was confirmed in the PDCs by genomic profiling (data not shown). These results demonstrated that BD-138T retained the urothelial features and a genomic/molecular phenotype similar to those of the primary tumor.

**Figure 4:**
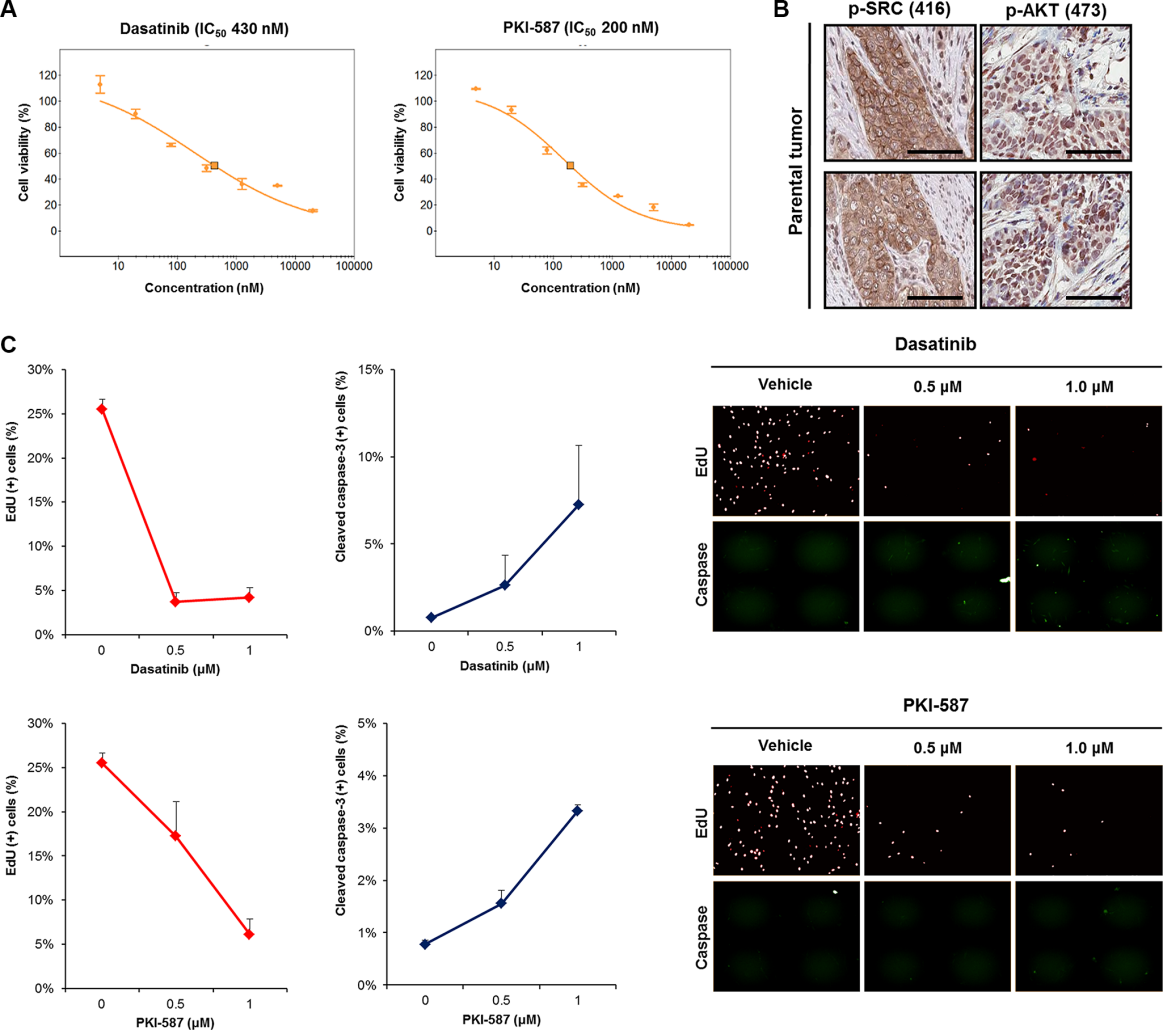
Anti-tumor efficacy of dasatinib (a SRC inhibitor) and PKI-587 (a PI3K/mTOR inhibitor) in 138T muscle-invasive bladder cancer harboring mutual EGFR amplification and PTEN deletion (**A**) Dose-response relationship curves of dasatinib and PKI-587 in 138T patient derived cell (PDC) tumors. Error bar represent the means ± SEM (triplicate). (**B**) Representative images of immunohistochemical staining of 138T PDC tumor tissue with antibodies against p-SRC (Tyr416) and p-AKT (Ser473). Scale bars, 100 μm. (**C**) 5′-ethynyl-2′-deoxyuridine (EdU) and cleaved caspase-3 analysis of 138T PDC tumors treated with dasatinib (Dose = 0, 0.5, or 1 μM) or PKI-587 (Dose = 0, 0.5, or 1 μM). Representative graphical analysis of EdU (+) and cleaved caspase-3 (+) cells following dasatinib or PKI-587 treatment; error bars represent the means ± SD. Representative images of EdU and cleaved caspase-3 staining following treatment (right).

After cytological confirmation, we subsequently screened the PDCs using a panel of anti-cancer agents (Table 1) considered particularly capable of addressing genomic alterations in bladder cancer. The BD-138T PDCs showed ample responses to a dual PI3K/mTOR inhibitor, PKI-587 (IC_50_ = 200 nM) and a SRC inhibitor, dasatinib (IC_50_ = 430 nM) (Table 1 and Figure 4A) whereas neither the agents targeting EGFR (erlotinib, gefitinib, lapatinib, and BIBW2992) nor those targeting mTOR (temsirolimus and everolimus) exhibited such effects (Table 1). In addition, BD-138T parental tumor tissues showed significantly activated SRC and AKT, suggesting these pathways as druggable targets specific to concomitantly *EGFR*-amplified and *PTEN*-deleted MIBCs (Figure 4B). BD-138T cells showed profoundly decreased mitotic fractions and an increased rate of apoptosis after treatment with 0.5 and 1 μM dasatinib or 0.5 and 1 μM PKI-587 (Figure 4C). Taken together, these *in vitro* data suggest that the combination of *EGFR* gene amplification and *PTEN* deletion in MIBC renders the cells sensitive to treatment with dasatinib and PKI-587 owing to upregulated SRC and AKT signaling pathways.

**Table 1:** *In-vitro* sensitivity of 138T PDC cell to a panel of targeted therapeutic agents^a^

Name	Mode of action	IC_50_ (nM)
Erlotinib	EGFR inhibitor	3800
Gefitinib	EGFR inhibitor	8600
Lapatinib	EGFR/HER2 inhibitor	11000
BIBW2992	EGFR/HER2 inhibitor	2600
Temsirolimus	mTOR inhibitor	8700
Everolimus	mTOR inhibitor	8500
PKI-587	PI3k/mTOR inhibitor	200
BEZ235	PI3k/mTOR inhibitor	660
Dasatinib	Abl/SRC/c-Kit inhibitor	430
Sorafenib	Raf-1/B-Raf/VEGFR-2 inhibitor	2700
AZD6244	MEK1 inhibitor	> 20000
Imatinib	v-Abl/c-Kit/PDGFR inhibitor	7200
Cabozantinib	VEGFR2/c-Met/Ret/Kit/Flt-1/3/4/Tie2/AXL inhibitor	4300
BAY73-4506	VEGFR1/2/3/PDGFRβ/Kit/RET/Raf-1 inhibitor	3300
ABT-888	PARP1/2 inhibitor	> 20000
Crizotinib	c-Met/ALK inhibitor	2400
INCB28060	c-Met inhibitor	> 20000
LY2835219	CDK4/6 inhibitor	4600
GDC-0449	Hedgehog/smothen inhibitor	> 20000
Gemcitabine	DNA synthesis inhibitor	4700
Oxaliplatin	DNA synthesis inhibitor	> 20000
Carboplatin	DNA synthesis inhibitor	> 20000
Irinotecan	Topoisomerase I inhibitor	8800
Etoposide	Topoisomerase II inhibitor	7900
Vinblastin	Microtubule inhibitor	1600
Pacilitaxel	Microtubule polymer stabilizer	> 20000
Docetaxel	Microtubule polymer stabilizer	> 20000

a IC_50_ is an average value derived from triplicated experiments.

### *In vivo* antitumor activity of dasatinib and PKI-587 in BD-138T MIBC PDXs

**Figure 5:**
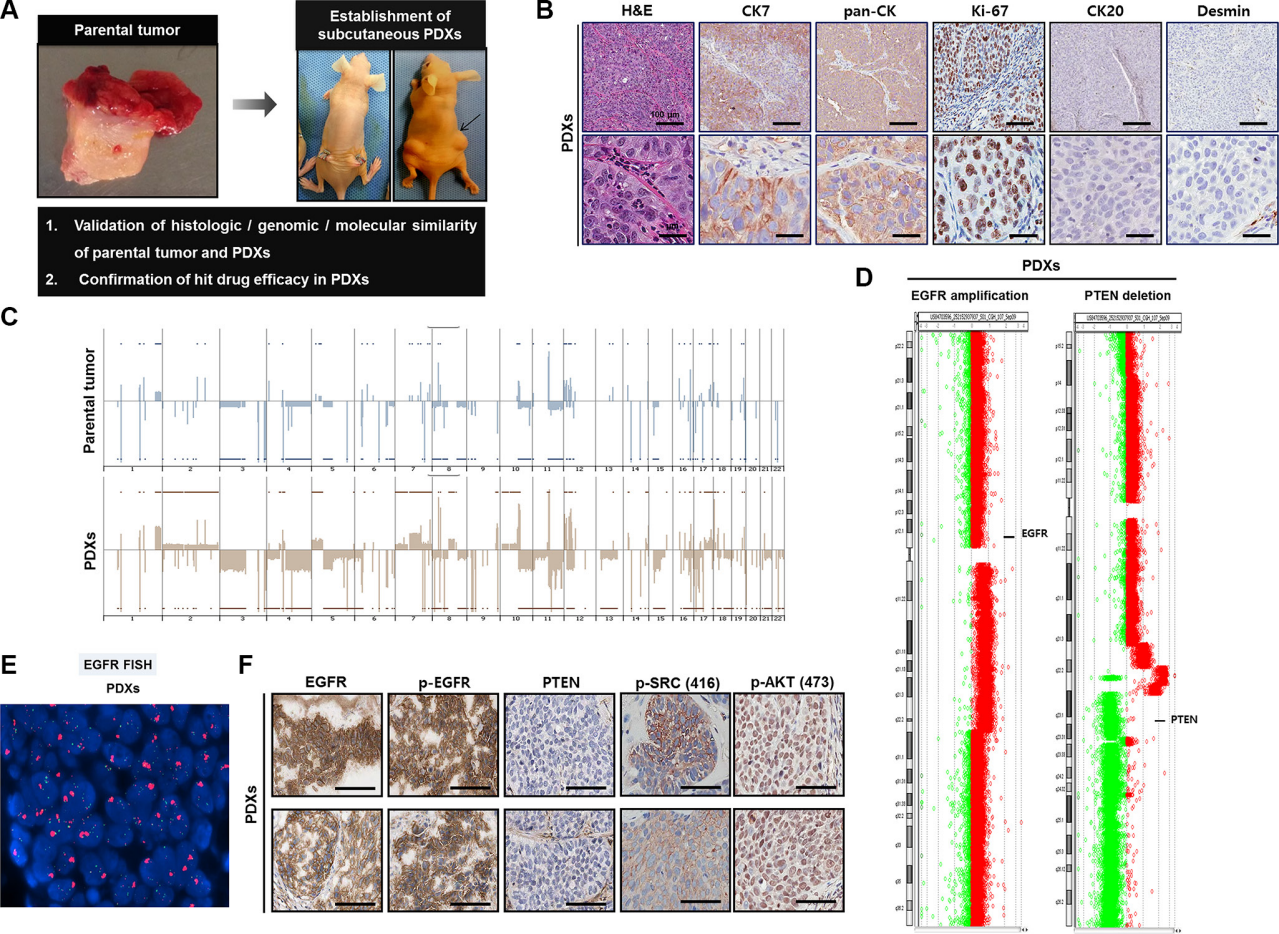
Establishment of patient-derived xenograft (PDX) models in 138T muscle-invasive bladder cancer (MIBC) and validation of the genetic, molecular, and histologic similarity between parental and PDX tumors (**A**) Schematic of the establishment of PDX tumors in 138T MIBC tissues. A PDX tumor was established by subcutaneous implantation in a BALB/c-nu mouse to validate the histologic, genomic, and molecular similarity of parental and PDX tumors. (**B**) Representative images of hematoxylin & eosin (H&E) and immunohistochemical (IHC) staining of 138T PDX tumor tissues with the indicated antibodies. CK7, cytokeratin 7; pan-CK, pan-cytokeratin; CK20, cytokeratin 20. Scale bars, 100 μm (Top), 20 μm (Bottom). (**C**) Array comparative genomic hybridization (CGH) analysis of 138T parental and PDX tumors. Gene amplifications and deletions were analyzed and compared between tumors to validate genetic similarity. (**D**) Array CGH analysis of 138T PDX tumors, illustrating the mutual *EGFR* amplification and *PTEN* deletion in the PDX and parental tumors. Individual chromosome ratio plots are shown with red representing the amplified region and green representing the deleted region. High-level *EGFR* amplification in chromosome 7 (left). *PTEN* deletion in chromosome 10 (right). (**E**) *EGFR* amplification assessed by FISH analysis in the 138T PDX tumor. Orange signal: *EGFR*; green signal: *CEP7*; blue signal: DAPI counterstaining. (**F**) Representative images of IHC staining of 138T PDX tumor tissue with EGFR, p-EGFR (Tyr1068), PTEN, p-SRC (Tyr416), and p-AKT (Ser473) antibodies. Scale bars, 100 μm.

To further verify our findings from *in vitro* studies and test the efficacy of targeting SRC and the PI3K/AKT/mTOR axis for MIBC therapy, we established subcutaneous BD-138T PDXs (Figure 5A). Microscopic examination through IHC analysis revealed the retention of morphological characteristics and a close correlation of the expression of various proteins between patient tumors and their corresponding PDXs, confirming that the tumor cells in the PDXs were indeed of human urothelial cell origin (Figure 5B). Furthermore, both PDXs and the original tumors exhibited conserved genomic DNA alterations including both *EGFR* amplification and *PTEN* deletion (Figure 5C–5E) as well as EGFR, p-EGFR, PTEN, p-SRC, and p-AKT expression correlating with that of the parental tumor (Figure 5F). Thus, the BD-138T PDXs described reliably retained the specific genetic, molecular, and histo-pathological features of the original tumors, suggesting that our models are pertinent to investigate potential molecular targets and to test the efficacy of targeting agents.

**Figure 6:**
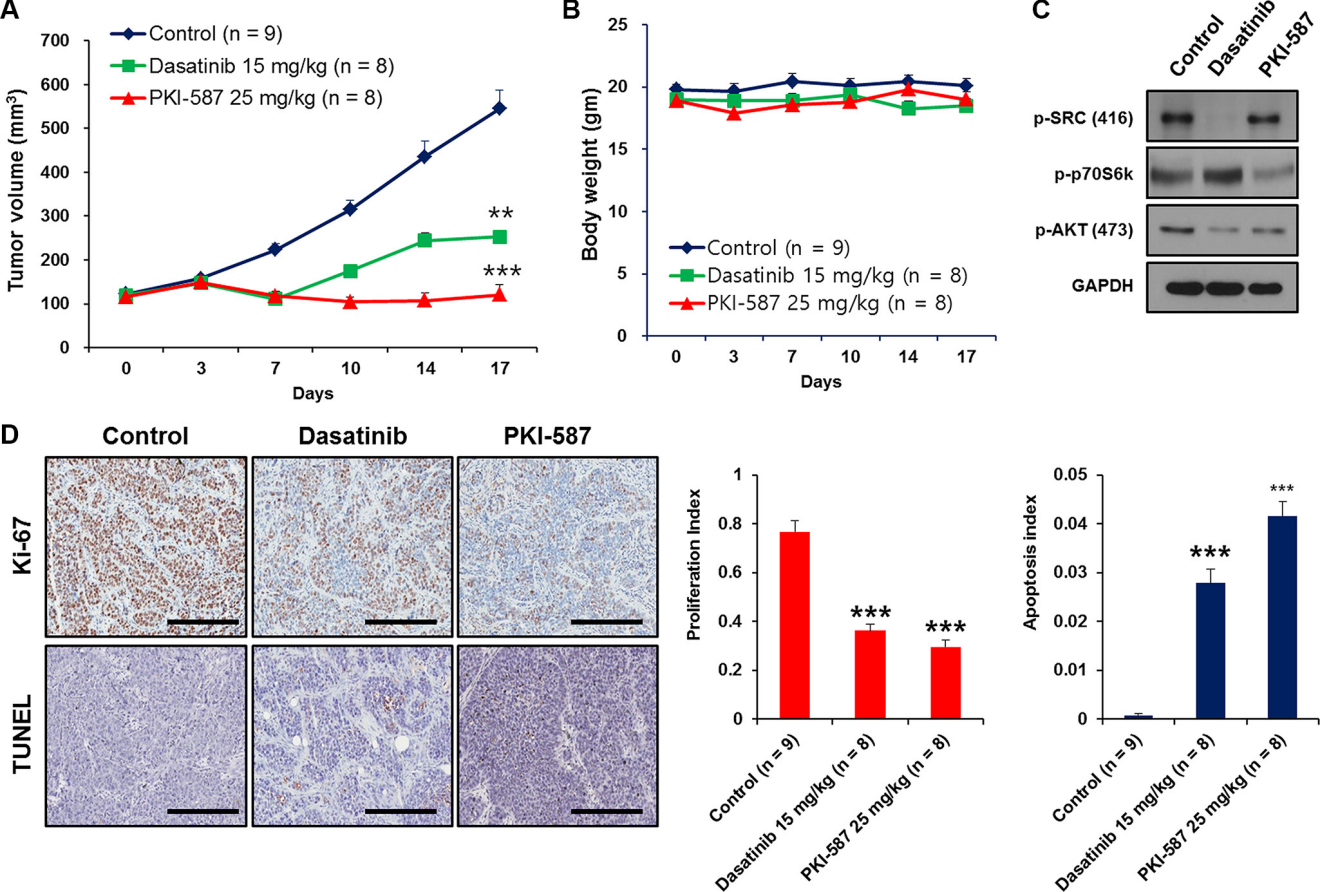
Dasatinib and PKI-587 inhibit tumor growth of 138T muscle-invasive bladder cancer with EGFR amplification and PTEN deletion in the patient-derived xenograft (PDX) tumor mouse model (**A**) Measurement of subcutaneous xenograft tumor size after treatment with dasatinib or PKI-587. **P < 0.01. ***P < 0.001. PDX tumors were treated with dasatinib (15 mg/kg, *n* = 8) and PKI-587 (25 mg/kg, *n* = 8) for 17 days, harvested, and processed for immunoblots. (**B**) Measurement of mouse body weight after treatment with dasatinib or PKI-587 showing no toxicity from the drug therapy in the PDX mouse model. Body weights were measured after treatment with dasatinib or PKI-587 for 17 days. (**C**) Immunoblots of p-SRC (Tyr416), p-p70S6K (Thr389) and p-AKT (Ser473) in xenograft tumors from (A). (**D**) Cell proliferation and apoptosis were measured immunohistochemically (IHC) by performing Ki-67 and TUNEL staining using PDX tissues treated with dasatinib or PKI-587 (left). Scale bar, 100 μm. Representative bar graphs of the IHC images (right), **P < 0.01, ***P< 0.001 (*n* = 5 fields; means ± SD, analyzed by two-tailed paired *t*-test).

As single agents, dasatinib (15 mg/kg) and PKI-587 (25 mg/kg) each showed strong anti-tumor activity *in vivo* in these models, with PKI-587 showing superior inhibitory effects on BD-138T tumor growth compared to dasatinib (Figure 6A). Single-agent treatment protocols were well tolerated by the mice, with no weight loss or other signs of acute or delayed toxicity (Figure 6B). As shown in Figure 6C, PKI-587 alone suppressed the phosphorylation of downstream effectors of PI3K/AKT/mTOR signaling (e.g., AKT at S473 and p70S6K at Thr389), whereas the biomarkers Tyr416-phosphorylated SRC and Ser473-phosphorylated AKT were observed in response to dasatinib treatment. These findings linked treatment with either dasatinib or PKI-587 to significant reduction of the mitotic fraction and significant induction of the apoptotic fraction (Figure 6D), consistent with the *in vitro* results. Together, these results suggest that BD-138T cells are sensitive to PKI-587 and dasatinib in xenograft tumors as well as *in vitro* and that this susceptibility is mediated through a blockade on proliferation and an increase in apoptosis.

## DISCUSSION

A substantial number of relapsed and metastatic MIBCs during the postcystectomy period are either already resistant to the nontargeted systemic chemotherapy at the time of initiation of systemic therapy or develop resistance to chemotherapy over time [2, 3, 19, 20]. The patient prognosis after radical cystectomy in MIBC depends on the extent of tumor invasion and whether lymph node invasion is present [21]. For patient BD-138T, whereas oncological follow-up was without indication of local relapse or distant metastasis at the time of this report, lymph node metastasis (N1) in the patient indicated that additional treatment to prevent the metastasis of bladder cancer to distant sites and to sensitize this patient to chemotherapy might be subsequently required.

Our findings indicate that although MIBC is genetically heterogeneous, therapies targeting specific gene alterations might be effective if patients with appropriate molecular profiles are identified prospectively. The finding that the PDX model shares the same genetic background as its donor patient tumor supports further evaluation of the patient-specific PDX platform for its ability to screen for the most effective tailored therapy to target the molecular drivers and for the most effective first-line and salvage chemotherapy agents for MIBCs. Previously, we developed and characterized a bladder cancer PDX model platform that incorporated 65 patient-derived tumor tissues with heterogeneous clinico-pathological features [13]. In the current study, to select anti-tumor drug candidates for a MIBC case harboring rare concomitant *EGFR* amplification and *PTEN* deletion, we used an integrative approach based on histopathologic and genomic characterization of the tumors, *in-vitro* drug HTS on PDCs and *in-vivo* validation of candidate drugs on PDXs. Previous studies suggested that MIBCs with an activated EGFR pathway were sensitive to EGFR inhibitors both *in vitro* and *in vivo* [22, 23]. In addition, PTEN-deficient tumors present an enhanced sensitivity to mTOR inhibitors owing to sustained activation of the PI3K/AKT signaling pathway [24]. As BD-138T contains both *EGFR* amplification and *PTEN* deletion, we originally predicted that one or more of the EGFR inhibitors or mTOR inhibitors might be effective agents against this tumor. However, agents targeting EGFR or mTOR did not elicit anti-tumor responses in the BD-138T PDCs. Subsequent preclinical experiments based on a platform that accurately mimics MIBC harboring mutual *EGFR* amplification and *PTEN* deletion demonstrated that the antitumor effects of dasatinib and PKI-587-based therapy manifest through the inhibition of SRC and PI3K/mTOR/AKT signaling.

Whereas EGFR mutations in urothelial carcinomas appear to be rare events, in the TCGA study there was a 9% incidence of *EGFR* amplification in 131 MIBC samples [6]. The level of EGFR expression directly correlates with tumor grade, stage, and survival in human bladder cancer [25–27], thus serving as a novel prognostic marker to predict the chemo-responsiveness of patients with locally recurrent or metastatic MIBC [28, 29]. Conversely, the clinical studies performed in patients with recurrent and metastatic MIBC using anti-EGFR treatments overall demonstrate a lack of efficacy [30–32]. Consequently, the identification of genetic alterations linked to primary response and the report of secondary genetic events leading to acquired resistance to anti-EGFR therapies still represent important diagnostic challenges.

The oncogenic activation of intracellular signaling downstream of EGFR including the RAS/RAF/MAPK, phospholipase C, PI3K/PTEN/AKT, and signal transducer and activator of transcription (STAT) pathways [33] represents a critical mechanism for resistance to EGFR inhibitors [34–36]. For example, as PTEN negatively regulates the PI3K/AKT pathway, it is easy to speculate that PTEN inactivation leads to EGFR inhibitor resistance [37]. It is well demonstrated that the PI3K/AKT/mTOR pathway is constitutively activated in a large number of MIBCs, primarily because of loss of heterozygosity or decreased expression of PTEN [38, 39], which is correlated with poor survival in MIBCs [40]. Notably, PTEN-deficiency in bladder cancer cells yielded disappointing results following rapamycin [41] or everolimus [42] treatment compared to cells expressing wild-type PTEN, likely because facilitation of AKT activation upon PTEN loss can have a more important role in driving the feedback loop in response to mTOR inhibition than in promoting the mTOR pathway. A more robust activity of novel dual PI3K/mTOR inhibitors, compared with either PI3K or mTOR inhibitors [43, 44], is required for the efficient suppression of tumor growth when multiple alterations of the PI3K pathway exist [45], in particular in the absence of functional PTEN. PKI-587, the most potent dual PI3K/mTOR inhibitor advanced to clinical development to date [46], suppresses the phosphorylation of PI3K/mTOR effectors and induces apoptosis in human cancer cell lines with elevated PI3K/mTOR signaling [47].

Despite playing a central role in multiple tumorigenic signaling networks of the SRC/FAK pathway [48, 49], SRC itself is rarely mutated in cancers [49]. Instead, SRC can be activated by receptor tyrosine kinases including EGFR, and SRC and EGFR act synergistically through mutual phosphorylation and activation [50, 51]. Studies have indicated that EGFR mutations and possibly other abnormalities of EGFR leading to enhanced SRC expression are important determinants of tumor sensitivity to SRC kinase inhibitors [52, 53]. Dasatinib is a novel and multi-targeted kinase inhibitor that targets important oncogenic pathways including SRC family kinases [53], demonstrating significant preclinical activities in tumors expressing active SRC-family kinase signaling [54, 55]. For example, the combination of a SRC inhibitor and an EGFR inhibitor synergistically enhanced apoptosis in EGFR-dependent lung cancer cells [56].

In summary, we demonstrated that inhibition of the PI3K/AKT/mTOR and SRC/FAK pathways utilizing PKI-587 and dasatinib exhibits anti-tumor effects in MIBC with concomitant *EGFR* amplification and *PTEN* deletion, thus providing additional potential treatment options through a precision medicine approach. Our results indicate that a library representing heterogeneous MIBCs would therefore likely represent a powerful preclinical platform to further develop personalized approaches for the treatment of patients with rare complex genomic alterations.

## MATERIALS AND METHODS

### Patient sample collection

This study was carried out in accordance with the principles of the Declaration of Helsinki, and was approved by the Samsung Medical Center (Seoul, Korea) Institutional Review Board (IRB) (no. 2010-04-004). The patient in this study provided written informed consent for the research. Fresh tumor tissue was obtained from bladder tumor tissue of the patient, diagnosed with MIBC with single lymph node metastasis, by surgical excision under sterile conditions. Surgical specimens were divided into three portions for implantation into immune-deficient BALB/c nude mice (Orient Bio, Seoul, Korea), DNA/RNA extraction, and pathologic assessment. The case was classified according to the 2016 WHO Classification system. For the latter, serial 5-μm sections from each formalin-fixed paraffin-embedded block were processed for H&E staining and examined by a specialized pathologist.

### Histologic analysis with IHC

For IHC analysis, each section of the paraffin-embedded tissue was blocked and permeabilized with 0.3% Triton ×-100 and 10% horse serum in PBS. Then, the tissue was stained with the following primary antibodies: anti-human CK7 (Dako Corp., Glostrup, Denmark), anti-human pan CK (Dako), anti-human CK20 (Dako), anti-human Desmin (Santa Cruz Biotechnology, Dallas, TX, USA), anti-Ki67 (Santa Cruz), anti-EGFR (Cell Signaling Technology (CST), Danvers, MA, USA), anti-pEGFR-Tyr1068 (CST), anti-PTEN (DAKO), anti-pSRC-Tyr416 (CST), and anti-pAKT-Ser473 (CST). After washing, the slides were incubated with secondary antibodies for 1 h at room temperature (RT) and counterstained with hematoxylin (Sigma-Aldrich, St. Louis, MO, USA).

### aCGH

aCGH was performed using SurePrint G3 Human CGH 4 × 180 K Microarrays (Agilent Technologies, Inc., Santa Clara, CA, USA). Labeling and hybridization were performed according to the manufacturer's instructions. The dual-colored fluorescence signals were scanned using the Agilent Microarray Scanner and translated to log10 ratios using Feature Extraction software (Ver-11.0.1.1, Agilent). Purified DNA was labeled with Cy5-dUTP following the Agilent Oligonucleotide Array-Based CGH for Genomic DNA Analysis protocol (Ver-7.3, Agilent). Data were obtained using Agilent Feature Extraction Software 9 and analyzed with Agilent CGH Analytics Version 6.5 software, using the ADM-2 statistical algorithms with 6.0 sensitivity thresholds. Based on the aCGH data, the extracted signals were normalized to log2 ratios using the limma package [57]. We classified the segmented results into copy losses when the log2 ratios were lower than −0.25 and into copy gains when these were greater than 0.25.

## EGFR FISH

EGFR gene copy number was determined by FISH using the dual probe LSI EGFR Spectrum Orange/CEP7 Spectrum-Green Probe (Vysis; Abbott Laboratories, Abbott Park, IL, USA) according to the manufacturer's instructions and as previously described [34]. Normal EGFR/CEP7 signals (one to two copies per cell) in the various non-neoplastic cells served as the internal positive control. We defined EGFR gene amplification as an EGFR/CEP7 ratio > 2.0 in 20 tumor nuclei and polysomy-7 were regarded as negative for gene amplification. Metaphase analysis was performed on 20 consecutive spreads and interphase analysis was performed on 100 consecutive nuclei. Images were acquired using a cooled CCD camera (SenSys, Photometrics, Tucson, AZ, USA).

### Variant detection using a customized cancer panel (CancerSCAN^™^)

The Cancer Panel is a targeted next-generation sequencing assay that was developed, validated, and provided by the Samsung Genome Institute (Samsung Medical Center, Seoul, Korea) [18]. It includes all exons from 81 cancer-related genes and 31 introns from 5 genes recurrently rearranged in cancer. Using the Illumina HiSeq 2500 instrument (San Diego, CA, USA), the captured libraries underwent paired-end high-depth sequencing (target > 800 × coverage). Data were analyzed using an automated bioinformatics pipeline designed to detect various genetic alterations including single nucleotide variations, insertion and deletion, gene amplification and deletion, and gene fusions [58].

### Primary *in vitro* short-term culture of PDCs

PDC cultures were established through single cell dissociation of tumor specimens according to previously published protocols [59, 60]. Cell suspensions of the patient tumor were acquired through tissue dissociation utilizing a gentleMACs™ tissue dissociator (Miltenyi Biotech, Bergisch Gladbach, Germany) and the resulting suspensions were extruded through a 70-μm nylon filter. Extracted cells were cultured in neurobasal media-A supplemented with 10% FBS in a humidified atmosphere of 5% CO_2_ at 37°C. Authentication of these cells was performed using short tandem repeat (STR) profiling.

### Immunocytochemistry (ICC)

For ICC, tumor cells were fixed in 4% paraformaldehyde (PFA) in PBS for 20 min and blocked with 5% donkey serum. Then, they were incubated with primary antibodies including anti-human CK7, anti-human Pan-CK, anti-human CK20, and anti-human Desmin for 16 h at 4°C and then with anti-mouse Alexa 594 (Invitrogen, Carlsbad, CA, USA) secondary antibodies for 2 h at RT with washing in between. Microscopy analysis was performed using a confocal microscope (LSM700, Zeiss, Oberkochen, Germany).

### *In vitro* HTS drug sensitivity assay

Primarily dissociated BD-138T MIBC cells were seeded in 384-well plates at 500 cells per well. At 2 h after plating, cells were treated with a drug library in 3-fold and 10-point serial dilution series (*n* = 3 for each condition). The cells were incubated at 37°C in a 5% CO_2_ humidified incubator for 6 days and cell viability was analyzed using an ATP monitoring system based on firefly luciferase (ATPlite 1 step; Perkin Elmer, Waltham, MA, USA) according to the manufacturer's protocol. The drug library was composed of a variety of targeted and cytotoxic agents (erlotinib, gefitinib, lapatinib, BIBW2992, temsirolimus, everolimus, PKI-587, BEZ-235, dasatinib, sorafenib, AZD6244, imatinib, cabozantinib, BAY73-4506, ABT-888, crizotinib, INCB28060, LY2835219, GDC-0449, gembitabine, oxaliplatin, carboplatin, irinotecan, etoposide, vinblastin, paclitaxel, and docetaxel; all purchased from Selleck Chemicals, Houston, TX, USA). The drugs were prepared and diluted according to the manufacturer's instructions. IC_50_ values were calculated as an average of triplicate experiments using the S+ Chip Analyzer (Samsung Electro-Mechanics Company, Ltd., Gyeonggi, Korea) [61].

### Evaluation of *in vitro* drug effects on cell proliferation and apoptosis

To quantitatively access the fraction of PDCs undergoing apoptosis or proliferation by dasatinib and PKI-587 *in vitro*, we used an automated high-content screening system (Operetta, Perkin Elmer). PDCs were seeded prior to drug treatment in 384-well black wall microplates in the culture medium at 500 cells per well. The cells were incubated for 24 h to allow cell attachment to the substrate and then caspase-3/7 detection reagent (1 μM, Molecular Probes, Eugene, OR, USA) was added to the samples. Dasatinib (0.5 and 1.0 μM) and PKI-587 (0.5 and 1.0 μM) were then applied separately on triplicate samples. A day before sample fixation, cell culture medium containing 5-ethynyl-2′-deoxyuridine (EdU, 10 μM) compound from the Click-iT EdU imaging Kits (Molecular Probes) was added to the samples followed by 24 h incubation to allow EdU incorporation into the DNA of cells undergoing active DNA synthesis (proliferation). For counterstaining of the nuclei and detection of casepase-3/7 and EdU, the cells were fixed using PFA (4% w/v in PBS) for 20 min, permeabilized with Triton ×-100 (0.15% v/v in PBS) for 20 min, and blocked in BSA (3% w/v in PBS) for 1 h at RT. Then, the Click-iT reaction cocktails, prepared according to the manufacturer's recommendation, were applied to the samples and allowed to react for 1 h at RT. After washing three times with PBS, DNA staining was performed using Hoechst33342 (diluted in 3% BSA at the final concentration of 10 μg/mL) for 30 min, also followed by three washes with PBS. Fluorescent signals were automatically acquired with the Operetta system and then the data were analyzed using Harmony software (PerkinElmer, Waltham, MA, USA). The number of live cells in each well was determined depending on the size, roundness, and intensity of DNA and caspase-3/7 staining to discriminate intact nuclei without showing nuclear condensation, fragmentation, and caspase activation, respectively. The percentages of caspase-3/7+ or EdU+ cells reflect the nuclei showing fluorescence signals (Alexa Fluore488 for caspase-3/7 and Alexa Fluor594 for EdU) greater than the threshold level set by considering the background fluorescence level of each channel.

### *In vivo* efficacy studies in established MIBC PDXs

Animal experiments were carried out in accordance with the Institute for Laboratory Animal Research Guide for the Care and Use of Laboratory Animals and following protocols approved by the IRB at the Samsung Medical Center (No. 2014-11-19-003). Briefly, Matrigel (BD Biosciences, Bedford, MA, USA)-embedded tumor fragments (1–2 mm^3^) were directly implanted into the subcutaneous pockets made in one side of the lower back of BALB/c nude mice (female, 6–8 weeks old; Orient Bio) as previously described [60]. Some PDX tumors were harvested and divided into two samples for DNA/RNA extraction and histopathologic examination. The origin of each xenograft was validated by STR DNA fingerprinting.

For *in vivo* drug efficacy testing, calipers were used to measure the tumor size and tumor volume was calculated according to the formula (a × b^2^)/2, where “a” was the longest diameter and “b” was the shortest diameter of the tumor. Mice bearing established subcutaneous PDXs (100–150 mm^3^) were randomly allocated to three groups to receive the following: vehicle (*n* = 9); dasatinib 15 mg/kg i.p. on a daily dosing regimen (*n* = 8) for up to 17 days [62, 63]; or PKI-587 25 mg/kg i.v. once a week for 17 days (*n* = 8) [47, 64]. Throughout the study, the mice were weighed and the tumor burden was monitored every 3 days. Mean tumor volumes were calculated and growth curves were established as a function of time. After 17 days of treatment, animals were sacrificed, the tumors were explanted, and the tumor tissue was dissected and snap-frozen using dry ice for further processing to use in western blotting (WB) and IHC. To decipher the signaling pathway relevant to the antitumor effect of dasatinib and PKI-587 on BD-138T cells, tumor tissue was isolated from BD-138T xenografts at the end of the treatment course and analyzed by WB. The effects of dasatinib and PKI-587 on cell proliferation and the extent of tumor apoptosis were assessed by IHC staining of human Ki-67 (Zymed Laboratories, San Francisco, CA, USA) and terminal deoxynucleotidyl transferase-mediated nick-end labeling (TUNEL) assays, respectively. The TUNEL assay was performed as previously described [65] using the DeadEnd Fluorometric TUNEL System (Promega, Madison, WI, USA) according to the manufacturer's instructions. All slides were also counterstained with Mayer's hematoxylin. The proliferative or apoptotic index was calculated as a ratio of the Ki-67-positive or TUNEL-positive cell number to the total cell number in high-power (x400) fields.

### Preparation of protein extracts and WB

For WB analysis, tumors were lysed with ice cold RIPA buffer supplemented with a protease inhibitor cocktail and phosphatase inhibitors (Roche Diagnostics, Roswell, GA, USA). Lysates were clarified by centrifugation at 13,000 rpm for 30 min, after which the supernatants were harvested. Protein concentrations were determined using a bicinchoninic acid protein assay kit (Thermo Fisher Scientific, Waltham, MA, USA). Equal amounts of protein were subjected to SDS-PAGE and transferred to polyvinylidene difluoride (PVDF) membranes (Millipore, Billerica, MA, USA). After blocking nonspecific binding sites on the membranes with 5% skim milk or 5% BSA for 2 h at RT, the membranes were incubated with the indicated primary antibodies overnight at 4°C and then with the appropriate secondary antibodies for 1 h at RT. Antibodies against pSRC-Tyr416 (CST), p-p70S6K-Thr389 (CST). pAKT-Ser473 (CST), pErK(1/2)-Thr202/Tyr204 (CST), and GAPDH (CST) were used. Immunoreactive bands were visualized with HRP-conjugated secondary antibodies and a chemiluminescent substrate by exposure to X-ray film.

### Statistical analysis

The data are shown as averages and standard deviations. Two-tailed Student's *t*-tests were used to compare the data. All *P*-values were two-sided and a *P* < 0.05 was considered statistically significant. All data analyses were performed with SPSS statistical software (Statistical Product and Services Solutions, version 19.0, Chicago, IL, USA).
